# Getting on your last nerve: IFNs and resistance to infection

**DOI:** 10.1093/jimmun/vkag169

**Published:** 2026-06-30

**Authors:** Molly E Bunkofske, Christopher A Hunter

**Affiliations:** Department of Pathobiology, School of Veterinary Medicine, University of Pennsylvania, Philadelphia, PA, United States; Department of Pathobiology, School of Veterinary Medicine, University of Pennsylvania, Philadelphia, PA, United States

**Keywords:** neuron, IFN, STAT, MHC-I

## Abstract

Interferons (IFNs) affect almost all nucleated cells and induce transcriptional events that are associated with control of intracellular infections as well as regulatory pathways that influence inflammatory processes. How different hematopoietic and non-hematopoietic cell types interpret these signals is a reflection of cellular context and function. For example, neurons are often viewed as immunologically inert and have muted responses to IFNs. However, because neurons can be targeted by several classes of pathogens, there is a need for these cells to sense infection and respond to IFNs to engage anti-microbial activities. This review discusses the unique features of neuronal IFN signaling, emphasizing how JAK-STAT pathway engagement and downstream induction of interferon-stimulated genes (ISGs) shape intrinsic and extrinsic neuronal anti-pathogen defense.

## Introduction

Interferons (IFNs) are a group of cytokines, broadly conserved across vertebrate species, that orchestrate host defense against intracellular pathogens through the induction of antimicrobial states and modulation of the immune response. Originally named for their ability to “interfere” with viral replication, IFNs were initially described as a soluble factor produced by virus-infected cells that “interfered” with subsequent viral infection.^1^ This antiviral activity was shown to confer broad protection rather than virus-specific immunity and established IFNs as a critical component of innate host defense (reviewed in^2^). It is now well-established that IFNs can act in an autocrine and paracrine fashion to limit or clear infection. These cytokines can be broadly classified into three types based on structure, cellular source, and function: type I IFNs (>10 mammalian subtypes, but primarily IFN-α, IFN-β), a single type II IFN (IFN-γ), and type III IFNs (>2 subtypes of IFN- λ). Each of the IFNs engage structurally similar yet distinct receptors that converge on Janus kinase (JAK)-signal transducer and activator of transcription (STAT) signaling pathways to drive expression of interferon-stimulated genes (ISGs). Type I IFNs are produced by most cell types and are important for antiviral defense whereas type III IFNs are most often produced at barrier sites by epithelial cells. While type III IFNs are produced within the central nervous system (CNS) in response to infection (reviewed in^3^) little is known about their role in the brain and these will not be discussed further. In contrast, IFN-γ is secreted by natural killer (NK) and T cells and this provides a mechanism that allows these lymphocytes to amplify the IFN response to infection.

IFNs exert broad and pleiotropic effects within the CNS that influence a number of biological processes from pathogen defense to blood-brain barrier integrity, synaptic function, and behavior (reviewed in^4^).^5^ For several infections that directly impact the CNS, IFNs mediate local resistance to these neurotropic viruses (reviewed in^6^^,^^7^). In addition to infection-induced IFNs, the CNS is exposed to low-level tonic type I IFN activity under homeostatic conditions (reviewed in^8^), which may prime innate immune readiness but also shape neuronal responsiveness.^9^^,^^10^ However, dysregulated or sustained IFN signaling in the CNS is the basis for type I interferonopathies that can drive immune-mediated tissue damage and dysfunction (reviewed in^11^). This balance between protective and pathological responses highlights the need for mechanisms that allow coordinated IFN responses to promote effective pathogen control while constraining excessive immune responses that threaten neural integrity.

**Figure 1:**
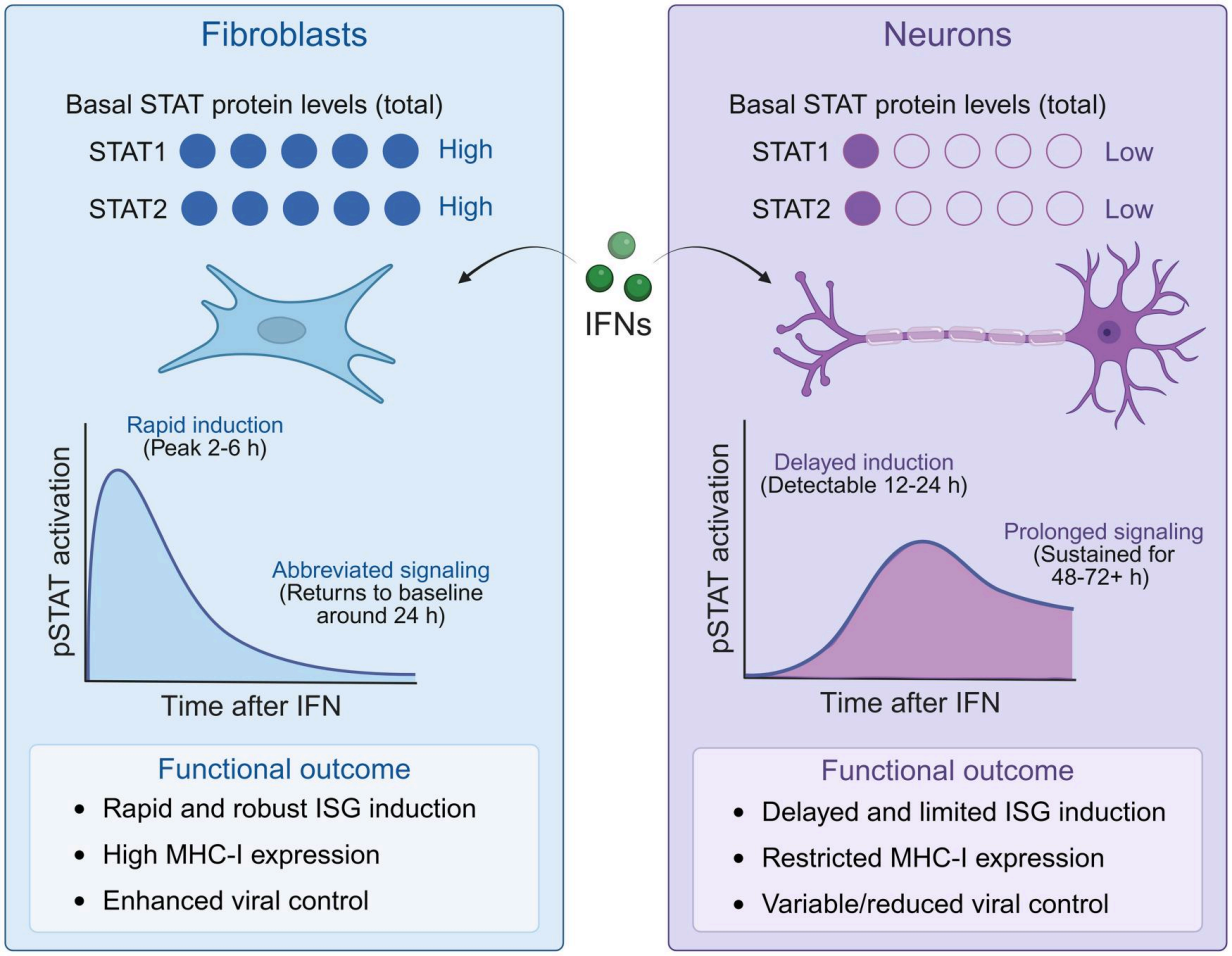
Neurons exhibit delayed but sustained responses to IFNs. Canonical type I/II IFN responses in fibroblasts (left) are contrasted with the distinct signaling dynamics observed in neurons (right). Fibroblasts express high basal levels of STAT1/2, enabling rapid IFN responsiveness characterized by early, robust STAT phosphorylation (pSTAT) followed by efficient signal termination. In contrast, neurons express low basal levels of STAT1/2 and require time to upregulate signaling components (eg STATs, JAKs) before mounting a detectable response. Consequently, neurons exhibit delayed pSTAT kinetics with peak activation occurring later but is sustained rather than transient. These distinct neuronal signaling properties are associated with a delayed and restricted interferon-stimulated gene (ISG) program, including MHC-I upregulation, and variable or reduced antiviral control. Figure created using BioRender.com.

While IFN-STAT signaling has been extensively characterized in immune cells, non-hematopoietic cells are also important participants in the IFN response during infection. Since somatic cells are susceptible to a large number of intracellular pathogens, it is essential to have mechanisms that allow these cells to control infection. Thus, IFNs have a key role in multiple mechanisms that act to limit microbial replication in these cells which include death of infected and bystander cells. The consequences of these inflammatory processes are not typically catastrophic because many cell types and tissues are capable of renewal. In contrast, neurons of the mature CNS are exceedingly long-lived, generally non-renewable cells that receive, process, and transmit electrical signals throughout the brain to influence cognition and behavior. Given the importance of maintaining the integrity of the brain, it has been proposed that neurons utilize specialized regulatory mechanisms to limit interactions with the immune system and mitigate the widespread loss of neurons that would profoundly affect the ability of a host to function. This may help explain why neuronal responses to IFNs (characterized by delayed induction and extended duration of STAT signaling) appear distinct from other cell types (Fig. 1). Given that MHC-I expression is a downstream consequence of IFN signaling and required for CD8^+^ T cell recognition, the low or absent MHC-I levels on neurons supports the idea that distinct rules govern how these cells interface with and are surveilled by the immune system.

One downside to strategies that protect neurons from the immune system is they may enable the establishment of persistent infections within the CNS. Originally thought to be immunologically inert, neurons have been viewed as a passive target for neurotropic pathogens (reviewed in^12^) While many neuroinvasive pathogens can infect multiple cell types (astrocytes, microglia, oligodendrocytes) in the CNS, neurons appear to be preferential host cells for long-term persistence. For example, they are a major reservoir for latent infection with herpes simplex virus (HSV) and varicella-zoster virus (VZV), while the parasite *Toxoplasma gondii* forms a quiescent cyst predominantly in neurons (reviewed in^13^). Multiple other viruses have been documented to infect neurons and establish persistent CNS infections, including: measles virus (MV), West Nile virus (WNV), rabies virus, cytomegalovirus (CMV), and Zika virus (reviewed in^13^) However, neuronal ability to produce and respond to IFNs highlights their role as active participants in pathogen control, while their ability to present MHC class I restricted antigens allows them to interact with CD8^+^ T cells. This review describes the canonical IFN signaling pathways, mediated by type I and type II IFNs, their unique regulation in neurons, and the functional consequences of these adaptations during infection. In addition, we highlight recent insights into how IFN-γ can facilitate neuron-specific interactions with CD8^+^ T cells to help mediate pathogen control.

### Neuronal sensing of infection and production of type I IFNs

The production of type I IFNs in the CNS is a hallmark of many infections and an important process for local pathogen control (reviewed in^14^). Expression of type I IFNs is initiated through the sensing of pathogen-derived ligands (or PAMPS) by pattern recognition receptors (PRRs), which activate the transcriptional pathways necessary for IFN-α/β production. In vitro studies involving stimulation with the PRR ligand poly(I: C) (a synthetic double-stranded RNA) or viral infection have consistently demonstrated that neurons can produce type I IFNs.^15–24^ However, the lack of extensive comparative studies means it is unclear to what extent the ability to produce these cytokines compares with other cell types or differs amongst neuronal subtypes. Nevertheless, neurons of the CNS have been shown to express toll-like receptors (TLRs), a set of PRRs expressed either on the cell surface or within endosomes that recognize distinct PAMPS: these include TLR2 (lipoproteins), TLR3 (dsRNA), TLR4 (LPS), TLR7/8 (ssRNA), and TLR9 (CpG) (reviewed in^25^). There are also cytoplasmic sensors that detect the presence of PAMPS or infection-induced changes. Cortical neurons have been shown to utilize MDA5/RIG-1 to respond to poly(I:C) stimulation^21^ and RIG-1 to detect infection with Japanese encephalitis virus (JEV).^26^ Thus, rather than passive targets of infection, these types of observations position neurons as competent sensors of infection, capable of initiating and contributing to innate immune responses in the CNS.

### IFNs and JAK-STAT signaling in neurons

Canonical IFN signaling through the JAK-STAT pathway is a central driver of ISG expression and defining how these pathways operate in neurons is helpful to understand how the immune system might operate in the CNS. Type I IFNs initiate this pathway by binding to the heterodimeric IFN-α/β receptor (IFNAR) composed of IFNAR1 and IFNAR2, which are ubiquitously expressed by nearly all cells and associated with the kinases JAK1 and TYK2, respectively. IFN-γ binds to the IFN-γ receptor (IFNGR), which is composed of IFNGR1 and IFNGR2 subunits which engage with JAK1 and JAK2. Ligation of these receptors results in a series of phosphorylation events that converge on the phosphorylation of STAT1 (pSTAT1) which allows it to homodimerize and translocate to the nucleus where it binds to gamma-activated sequences (GAS) within target gene promoters to initiate transcription of ISGs. Type I IFN signaling can also phosphorylate STAT2 (pSTAT2) and lead to the formation of a transcriptional complex composed of STAT1–STAT2–IRF9, which binds to interferon-stimulated response elements (ISREs) within target genes to drive expression of a distinct set of ISGs. A key feature of canonical IFN signaling is that peak STAT activation and ISG induction is transient and abbreviated by negative feedback mechanisms that curtail JAK-STAT activity at the receptor, cytoplasmic, and nuclear level. Thus, IFN receptor availability is reduced through the removal of surface bound IFN receptors via endocytosis and these receptor: ligand complexes are either recycled or degraded (reviewed in^27^). A second termination mechanism involves the induced expression of the SOCS protein family (eg SOCS1 and SOCS3), which bind to and inhibit JAK1/2 and TYK2, preventing further STAT phosphorylation. The activity of signaling molecules is also controlled through a mechanism of targeted modifications and degradation that include the dephosphorylation of activated STATs by protein tyrosine phosphatases (PTPs) (reviewed in^28^).

The broad impact of IFN signaling is illustrated by the number of cell types that are responsive to these cytokines and, depending on the context and cell type, there are 50–2,000 ISGs that have been described (reviewed in^29^). Since type I and type II IFNs engage STAT1, there is significant overlap in the ISGs they induce, but each IFN type does trigger a unique subset of specific genes.^30^^,^^31^ Type I IFNs drive expression of ISGs that promote intrinsic antiviral defense and this includes the “classical ISGs” known to inhibit viral replication: PKR, Mx1, Oas1, ISG15, and Viperin (reviewed in^29^). In contrast, IFN-γ induces the expression of genes involved in antigen presentation (eg CIITA, NLRC5), immune cell recruitment (eg CXCL10), and macrophage activation (eg iNOS). Among the most prominent IFN-γ-induced effectors are the immunity-related GTPases (IRGs) and guanylate-binding proteins (GBPs), which are both families of GTPases that target and disrupt pathogen-containing vacuoles or directly attack cytosolic pathogens (reviewed in^32^).

There are numerous studies that demonstrate that neurons can respond to IFNs and activate downstream signaling. For example, the use of primary neurons and neuronal cell lines stimulated in vitro with IFN-α or IFN-β results in increased expression of STAT1/2 and various ISGs,^10^^,^^33–39^ while systemic administration of IFN-α in vivo increased STAT1 expression within neurons.^40^ In contrast, based on early studies, neuronal responsiveness to IFN-γ was characterized by a failure to induce downstream elements like IRF1 and MHC-I.^41–43^ It is now recognized that sustained exposure of neurons to IFN-γ results in increased expression of STAT1 and pSTAT1, the immunity-related GTPase (IRG) system, and MHC-I.^44–52^ These events are also observed in vivo in studies in which intraparenchymal injection of IFN-γ combined with transcriptional profiling of neurons showed increased expression of STAT1, guanylate binding proteins (GBPs), and MHC-I presentation machinery and that pSTAT1 and MHC-I expression were dependent on neuronal expression of IFNGR1.^53^

Although neurons possess the capacity to respond to IFNs, their responses appear distinct compared to other cell types (Fig. 1). In one study that compared the ability of murine embryonic fibroblasts (MEFs) and primary hippocampal neurons stimulated with IFN-β, MEFs exhibited a canonical STAT1/2 phosphorylation characterized by robust early but transient pSTAT1/2 levels whereas neurons had significantly lower levels of STAT1/2 activation, reduced expression of antiviral response genes, and an inability to restrict early LCMV infection.^10^ Similarly, when MEFs and primary hippocampal neurons were incubated with IFN-γ, neuronal activation of STAT1 was delayed and muted in magnitude associated with reduced expression of IFN-γ-responsive genes (CXCL10, IRF-1, SOCS-1).^54^ Surprisingly, in neurons, even after the removal of IFN-γ, pSTAT1 was maintained at a consistent level throughout the 48-h time course, likely due to extended JAK1/JAK2 activation.^50^ However, the observation that sustained STAT1 activation did not occur with lower concentrations of IFN-γ^49^ indicates that there is a high threshold for neurons to respond to IFN-γ.

**Figure 2:**
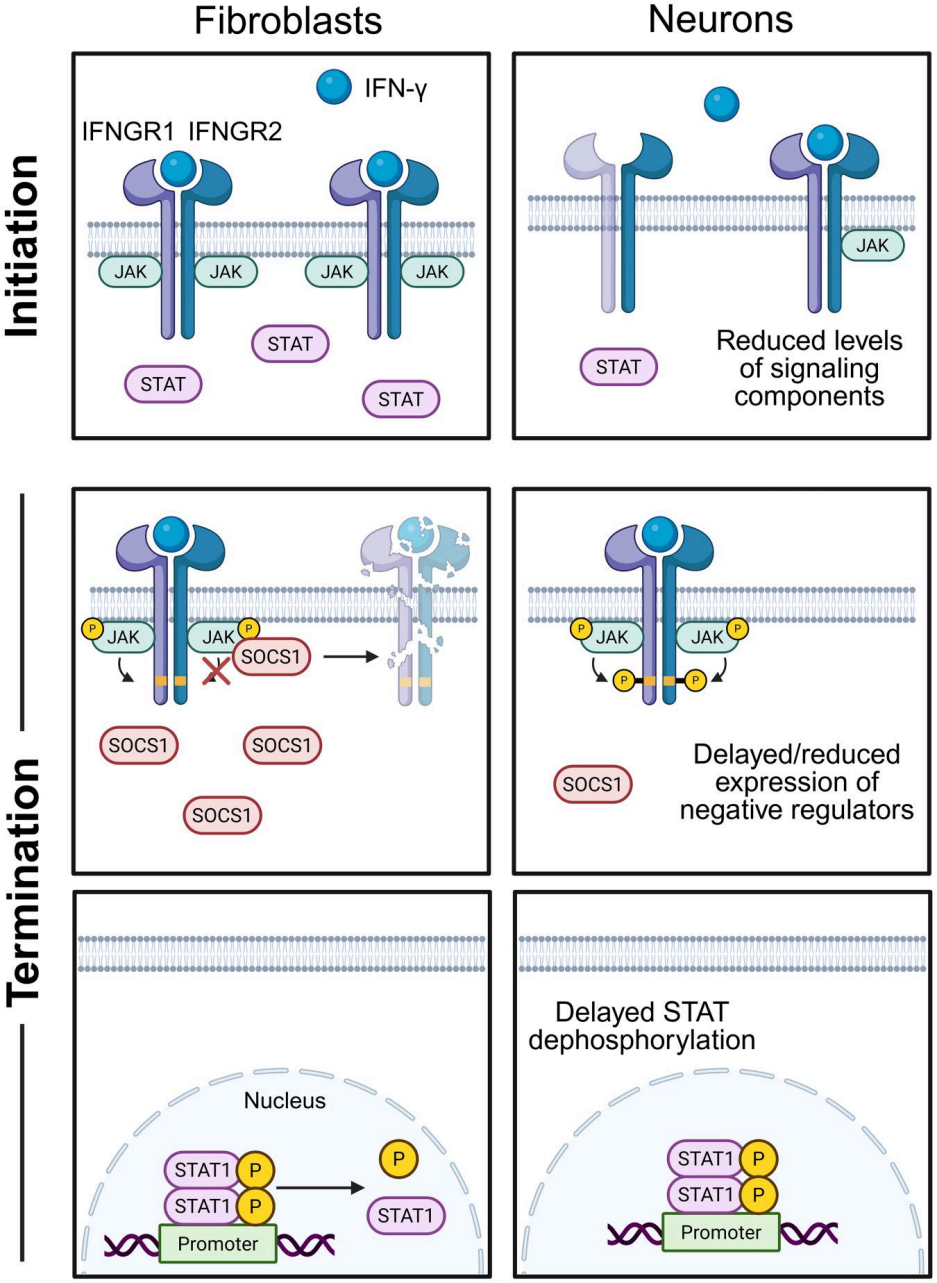
Mechanisms that underlie delayed/sustained STAT activation in neurons. Comparison of selected aspects of IFN signaling in fibroblasts (left) to neurons (right) that potentially contribute to delayed and sustained STAT signaling observed in neurons. Initiation (top) of IFN signaling in neurons may be delayed due to relatively low basal expression of IFN signaling components, including IFNGR1, JAKs, and STATs. Differences in termination (bottom) of STAT signaling, including sustained JAK activation due to reduced expression of SOCS1 and slower dephosphorylation of STAT1, may result in sustained STAT activation in neurons. Figure created using BioRender.com.

There are several possible mechanisms that may help to explain these differential STAT responses in neurons that include: (1) reduced IFN signaling machinery (2) extended JAK activation, and (3) reduced expression of negative regulators that lead to delayed STAT dephosphorylation (Fig. 2). IFNGR1 transcripts were found to be 8-fold lower in primary hippocampal neurons compared to MEFs,^54^ which may help explain the threshold of IFN-γ necessary to initiate JAK/STAT signaling. Similarly, while JAK1, STAT1, and STAT2 are constitutively expressed by MEFs and astrocytes, primary hippocampal neurons had lower levels of these signaling components but stimulation with IFN-γ significantly increased the levels of JAK1 and STAT1/2 proteins.^10^^,^^46^^,^^50^^,^^54^ These observations indicate that neurons require more time to increase their levels of signaling intermediates in order to induce downstream ISG expression. The report that JAK1/2 activation was extended in primary neurons^50^ and was necessary for prolonged STAT1 activation^49^ indicates that the IFN-γ receptor stays signaling-competent for longer within neurons. The dephosphorylation of STAT proteins is another mechanism that limits STAT activity and the ability of neurons to sustain pSTAT1 levels^50^ indicates that dephosphorylation of STAT1 occurs at a reduced rate. SOCS1 and SOCS3 are ISGs that are negative regulators of JAK1/2 and TYK2, and in IFN-γ-stimulated primary hippocampal neurons, SOCS1 (but not SOCS3) was expressed but delayed and maintained at low levels.^54^ It remains unclear, however, if these lower levels of SOCS1 contribute to sustained STAT activation.

The low basal expression and delayed activation kinetics of STATs could suggest that neurons prioritize STAT-independent pathways of IFN signaling. Indeed, IFNs also initiate PI3K and MAPK cascades which can influence antiviral responses, cell survival, metabolism, and stress responses (reviewed in^55^^,^^56^). Thus, in the absence of STAT1, primary neurons incubated with IFN-γ could still control MV.^57^ In one of the few studies on this aspect of IFN signaling in neurons, O’Donnell et al. found that IFN-γ-stimulated MEFs and primary hippocampal neurons rapidly (5 min) activated ERK1/2 but neurons induced five-fold greater activity that promoted neuronal cell survival.^46^ This contrast in kinetics between MAPK and JAK/STAT pathways remains an understudied aspect of IFN signaling in neurons. Collectively, these studies indicate that neurons are hardwired to have a muted yet sustained response to IFNs associated with differential regulation of the JAK-STAT cascade. This signaling pattern contrasts with the rapid, transient responses typical of immune cells and suggests that neuronal responses to IFN have been shaped by evolutionary pressure to balance defense with the need to preserve cell function.

### Functional outcomes of type I IFNs on neurons

The muted responsiveness of neurons to type I IFNs may help explain why certain viruses are able to persist within these cells and there are reports that document the inability of IFNs to mediate viral restriction within neurons in vitro. For example, the inability of IFN-β-treated primary neurons to restrict LCMV replication correlated with reduced expression of the antiviral genes Oas1a and ISG15.^10^ Similarly, pre-treatment of neuronal cell cultures with IFN-β did not limit the replication of TMEV or vesicular stomatitis virus (VSV) and this was associated with reduced expression of the ISG ApoL9b.^38^ Furthermore, differences in neuronal subtype (granule cell neurons vs. cortical neurons) expression of viperin, a key ISG that restricts replication of a range of RNA and DNA viruses,^58^ was reported to underlie differential susceptibility to infections with positive-stranded RNA viruses,^39^ yet how the neuronal subtypes compare in their responses to IFNs remains largely unexplored. Nevertheless, there are reports that primary neurons treated with type I IFNs can inhibit replication of positive strand viruses (WNV, SLEV, VEEV, and MHV),^39^ HSV-1, and VSV^59^ but this level of control is often modest. In contrast to many of the in vitro approaches, in vivo studies that utilize neuron-specific ablation of IFNAR or STAT1 clearly demonstrate that neuronal responsiveness to IFNs is important for efficient control of viral infections with HSV-1, VSV, and MHV.^59–62^ Such discrepancies between in vitro and in vivo models likely underscore the contribution of the CNS milieu to shaping neuronal defense. For example, in vivo there is evidence that there is homeostatic production of type I IFNs that maintains baseline expression of neuronal ISGs and this is hypothesized to prime them for antiviral defense.^9^^,^^10^ Thus, it seems unlikely that primary neurons derived from fetal tissues and cultured for days would recapitulate the experience in vivo of long-lived neurons that are exposed to tonic IFN signaling.

### Functional impact of IFN-γ on neurons

The production of IFN-γ by T cells within the brain can enhance anti-pathogen responses but also contribute to cell death and bystander damage as prolonged or excessive STAT1 signaling has been linked to deficits in behavior and neurotoxicity in the context of disease and infection (reviewed in^63^). The dampened and delayed response to IFN-γ observed in neurons could reflect a strategy adapted to limit such immune-mediated cytotoxicity within the CNS. Despite this, there is strong experimental evidence that IFN-γ promotes control of CNS infections with *T. gondii*, Sindbis virus (SINV), MV, VSV, polio virus type I, HSV-1, influenza, and JEV.^64–71^ There are even cases of IFN-γ exerting direct effects on infected neurons that includes the ability to restrict infection with *Listeria monocytogenes,*^72^ VSV,^51^ SINV,^65^ MV,^57^ and *T. gondii.*^48^

Although the exact mechanisms by which IFN-γ mediates pathogen control in neurons remain incompletely defined, studies that utilize the eukaryotic pathogen *T. gondii* have provided important insights. The lifecycle of *T. gondii* parasites includes tachyzoites that can infect neurons wherein they preferentially convert to latent stage bradyzoites and form long-lived intracellular cysts.^73^ IFN-γ is known to be important for parasite control within the CNS,^70^ but an early study concluded that neurons stimulated in vitro with IFN-γ do not control growth of *T. gondii.*^74^ More recent work has refined this view and demonstrated that sustained IFN-γ exposure enables human and mouse neurons to limit tachyzoite replication in an IRG-dependent manner.^48^ Consistent with this, in vivo evidence utilizing Cre-reporter fate mapping studies indicates that neurons infected with *T. gondii*^48^ or VSV^75^ can actually clear infection and survive. One explanation for these observations is that neurons exhibit delayed JAK/STAT signaling and low basal levels of pathway components, such that early IFN-γ exposure induces minimal ISG expression. Prolonged stimulation may therefore be required to build sufficient signaling capacity, allowing expression of ISGs such as Irga6, which targets the parasitophorous vacuole and promotes parasite clearance.^48^ In contrast, because viruses lack a defined vacuolar niche, IFN-γ–mediated control in viral infections is likely to rely on a broad ISG-driven antiviral state to block multiple stages of the viral life cycle.

As noted earlier, there are certain infections that have latent stages in neurons but little is known about whether the immune system is able to recognize and/or clear these persistent forms or prevent reactivation. IFN-γ has been shown to play a critical role in suppressing HSV reactivation in neurons,^76^^,^^77^ in part, by inducing autophagy-dependent antiviral mechanisms that limit viral gene expression and replication.^78^ For *T. gondii*, the loss of STAT1 in neurons in vivo results in increases in cyst number and size, indicating that IFN signaling acts on neurons to restrict the cysts.^79^ However, the ability of cysts to persist indicates that bradyzoites can evade clearance. This possibility is supported by reports that bradyzoites-derived effectors work together to limit STAT1 signals and antagonize IFN-γ–mediated necroptosis.^80–82^ It seems likely that this type of pathogen strategy to target IFN-mediated pathways will be a feature of other infections that naturally persist in neurons.

One of the major downstream effects of IFN-γ signaling in most cell types is the upregulation of MHC-I expression and enhancement of antigen presentation pathways. The ability of any infected cell to process antigen and load pathogen-derived peptides onto MHC-I molecules is required for interaction with CD8^+^ T cells, which can eliminate infected cells using cytotoxic means or enhance cell intrinsic defense through the local production of IFN-γ. The early finding that neurons lack basal MHC-I expression contributed to the belief that neurons are spared from CD8^+^ T cell surveillance thereby promoting viral persistence.^83^^,^^84^ Studies performed since then have demonstrated that steady state neurons do express low levels of MHC-I^85–88^ and that this expression is critical for non-immune functions like synaptic plasticity and brain development (reviewed in^89^) Nevertheless, neurons do upregulate MHC-I in response to treatment with IFNs, especially IFN-γ, and other cytokines, infection, and injury.^44^^,^^84^^,^^90–93^ As expected, given delayed STAT signaling kinetics, neurons treated with IFN-γ are slow to express surface MHC-I.^45^ There is evidence from in vitro studies that CD8^+^ T cells can recognize neurons infected with *T. gondii*, LCMV, Borna and picornaviruses.^94–98^ However, deletion of floxed MHC-I in neurons infected with VSV-Cre did not impact CD8^+^ T cell-mediated control of this infection.^75^ Thus, there remains a major knowledge gap in our understanding of the rules that govern when neuronal antigen presentation influences resistance or susceptibility to infection in vivo.

## Conclusions

There are numerous studies that support the concept that excessive inflammation or cytotoxic immune responses within the CNS can have pathological consequences for this tissue. Overexpression or sustained neuronal MHC-I expression has been linked to detrimental outcomes that include deficits in neurodevelopment and repair,^99^ as well as increased encephalitis and mortality during LCMV infection.^96^ Thus, the reduced and delayed induction of MHC-I by neurons may represent a mechanism to balance immune surveillance and mitigate the potential for T cell-mediated damage. Despite evidence that neurons can mount cell-intrinsic antimicrobial responses capable of restricting select intracellular pathogens, these cells still serve as reservoirs for latent infection and the mechanisms underlying this remain poorly understood. It is possible that the delay in neuronal responses to IFNs may provide a window for pathogens to survive and convert to latent forms that are more resistant to clearance mechanisms. Nonetheless, key questions remain regarding neurons and pathogen persistence, including: (1) What is the mechanistic basis for the extended IFN signaling within neurons? (2) Does this altered signaling support latency? (3) Can neurons mount responses that directly act on latent pathogens? (4) Are IFN signals to neurons sufficient to allow latently infected cells to be recognized by CD8^+^ T cells? As noted by others,^100^ there remains a need to develop better experimental models and tools to study latency that involve improved neuronal culture systems, in vivo imaging, and single-cell approaches which will be critical to dissect how IFNs regulate neuronal responses and how neurons support persistent infection. These types of studies should inform the development of strategies to enhance microbial control in the CNS without compromising neuronal integrity.

## Data Availability

This article is a review and does not report original data. No new datasets were generated or analyzed.
